# A review on aluminum matrix composites' characteristics and applications for automotive sector

**DOI:** 10.1016/j.heliyon.2024.e38576

**Published:** 2024-09-26

**Authors:** Xiaodong Wu, Wenkang Zhang

**Affiliations:** a Changsha Normal University, Changsha, 410100, China; b State Key Laboratory of Advanced Design and Manufacturing Technology for Vehicle, Hunan University, Changsha, 410082, China; c College of Engineering, Anhui Agricultural University, Hefei, 230036, China

**Keywords:** Aluminum matrix composites, Vehicle, Aluminum-plastic film, Mechanisms

## Abstract

In recent years, the auto industry has experienced significant advancements, making research and development (R&D) of vehicle materials increasingly vital. Aluminum matrix composites (AMCs), known for their lightweight, high strength, and excellent corrosion resistance, have demonstrated substantial potential in vehicle aesthetics, interior trim, power systems, and components manufacturing. Currently, aluminum-metal composites (such as Cu and Mg) and aluminum-nonmetal composites (including Si, C, and plastics) are the primary types of AMCs used in automobiles. A thorough investigation into their preparation, process mechanisms, and performance optimization is essential for the broader application of AMCs in new vehicle models. This review summarizes and analyzes the preparation methods, wear mechanism, performance enhancement strategies, strengthening mechanism, and economic impact of AMCs, discussing key influential factors to foster the development of new AMCs. Additionally, by examining the role of aluminum compound packing films in the pouch batteries of Electric Vehicles, also explores the future potential of AMCs within the new energy power sector.

## 1 Introduction

With the rapid advancement of society and technology, automobiles have become integral to daily transportation. However, the swift increase in the number of automobiles leads to environmental pollution, energy consumption, and safety issues. According to statistics, CO_2_ emissions could drop by 5 g/km for every 100 kg reduction in vehicle weight; furthermore, a 10 % decrease in vehicle weight corresponds to a 5.5 % reduction in electric consumption, a 5.5 % increase in range, and a 20 % decrease in daily costs [1,2]. Meanwhile, the development of vehicle materials is crucial for enhancing vehicle performance, reducing weight, ensuring passenger comfort, and improving safety. Aluminum (Al) is the most abundant metal in the Earth's crust, constituting about 8 % of the weight of the planet's solid surface. Al is extensively employed today due to its improved machinability, durability, ductility, malleability, and high strength-to-weight ratio. Currently, aluminum matrix composites (AMCs), known for their extremely low density and strong corrosion resistance, are popular lightweight materials. Casting components made from AMCs offer high quality, strong mechanical properties, and suitability for large-scale production. As a result, they are widely used in vehicle bodies, hubs, chassis, anti-corrosion beams, and battery cases to satisfy the needs and demands of the automotive industry [3]. As reported, the market is aiming to increase the aluminum content within a vehicle to reach 570 net pounds by 2030, and the cast aluminum alloy occupies more than 50 % of the total [4]. AMCs have emerged as a novel category of vehicle materials, attracting significant attention due to their lightweight properties, high strength, and excellent corrosion resistance. In the automotive manufacturing industry, AMCs are widely used in producing various components, such as vehicle bodies (including interior trim and exterior appearances) and critical systems like the suspension, chassis, and braking systems [5,6]. The choice of material for the vehicle body is particularly crucial for safety. Traditional materials like steel are characterized by considerable weight and suboptimal corrosion resistance, while AMCs effectively mitigate these limitations, thereby enhancing fuel efficiency and safety while contributing to the reduction of emissions. The engine, often referred to as the “heart” of the vehicle, directly affects its performance and economic efficiency. Conventional materials used in engine components, such as casting parts, have drawbacks like high density and low thermal conductivity. In contrast, AMCs offer superior thermal conductivity and strength. Utilizing AMCs in manufacturing cylinder blocks and heads can enhance engine efficiency, conserve energy, and thereby improve fuel efficiency, safety, and reduce emissions. The application of AMCs in the suspension system and braking system of automobiles is a hot issue. Traditional materials for automobile suspension system, such as steel, suffer high weight, poor corrosion resistance, and fatigue durability. In contrast, AMCs could avoid or alleviate problems of resisting corrosion from chemicals and humidity, thus enable automobiles to be handled better and be more comfort. In addition, AMCs feature flexible design, great plasticity, and sound processability, which enables them to meet requirements on complicated shapes and delicate structures. The demand for aesthetic appeal and variety in vehicle designs can be effectively met. However, AMCs present notable challenges, including high manufacturing costs, limited heat and abrasion resistance, and the necessity for specialized manufacturing processes and equipment. Moreover, the low recovery rates of AMCs pose a significant environmental concern. Therefore, the auto industry is currently facing increased demands for material performance. Corrosion and wear are significant concerns, as many components are made from metals that have low resistance to these issues, such as steel, aluminum, and magnesium. Lightweight materials are crucial for reducing greenhouse gas emissions and enhancing fuel efficiency, all while maintaining strength and durability. Another important aspect for the automotive manufacturing sector is the sustainability and recyclability of raw materials. Economic efficiency is vital for materials used in vehicle components, making the preparation method essential. Additionally, passenger safety remains the top priority. A comprehensive analysis of AMCs supports the exploration of their growing applications [[7], [8], [9], [10]].

This review examines the research advancements of key AMCs, such as Al-Mg, Al-Cu, Al-Si, Al-C, and Al-plastic composites, in automotive applications. Their fabrication methods, key parameters, strengthening mechanisms, wear mechanisms, and discussions on their applications are summarized and analyzed, aiming to provide a reference for extensive utilizing AMCs in automobiles.

## 2 AMCs

**Fig. 1:**
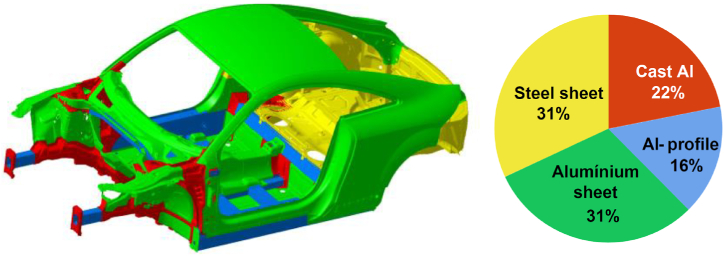
Aluminum-based materials application in automobile [9].

AMCs are composites made from aluminum and other materials, such as metal and non-metal, through a series of processing techniques. Compared with traditional steel, AMCs are superior in lightweight, high strength, and corrosion resistance [11]. Therefore, it is widely used in various parts of automobiles (Fig. 1). Based on compositions and performance characteristics, AMCs can be divided into aluminum metal (Cu and Mg) composites, aluminum non-metal (Si and C) composites, and Al-plastic composites. Besides, they can be classified into particle reinforcement, fiber reinforcement, and lamination by composite methods [[12], [13], [14], [15]]. Different types and application methods affect the mechanical properties, corrosion resistance, and service life of composite materials.

### 2.1 Al-metal composites

**Al-Cu composites:** with excellent conductivity, thermal conductivity, and formability, copper has been used extensively. However, the high cost of copper, and its density of ∼3 times higher than aluminum indicate that it is not favorable for the lightweight and cost-effecting manufacturing of auto parts. Thus, Al-Cu composites present huge application potential, because they could not only reduce the weight of the automobile structure, but also reduce production cost [[16], [17], [18]].

**Fig. 2:**
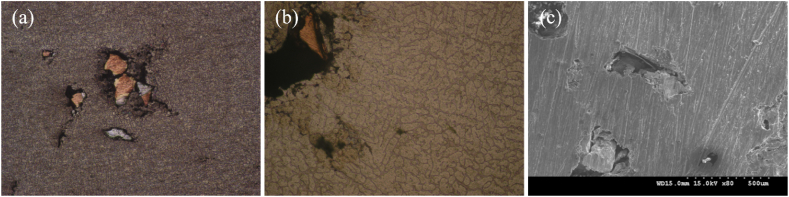
Surface morphologies of (a) Al-15 % Cu composite (100 × ), (b) the presence of void in composite (100 × ), (c) SEM image of voids in composite [27].

The preparation methods of Al-Cu composites include mechanical alloying, powder metallurgy, spark plasma sintering, vapor deposition, explosive bonding, and hot-pressing processes [[19], [20], [21]]. Among them, mechanical alloying is a commonly used method for preparing Al-Cu composites [22]. This method thoroughly mixes aluminum powder and copper powder, and then mills them in a high-energy ball mill to induce metallurgical reactions between metal powders, producing Al-Cu composite powders. Despite the simple operation and low cost, it shall accurately control the ball milling time and temperature when applying this method, so as to ensure the effective alloying reactions. Explosive cladding is an innovative technique for creating Al-Cu composites. The process works as follows: an explosive layer is placed between aluminum and copper plates, leading to rapid metallurgical reactions between the two metals due to the explosion. Consequently, alloy composites are formed [23]. This method produces high-strength composites within a short time, but it calls for the strict control of explosion conditions to ensure the quality of composites. The hot-pressing process triggers metallurgical reactions between aluminum and copper under the high temperature and high pressure to form the Al-Cu composites by stacking the two metal materials together [24]. This process is considered as a popular manufacturing method for composites due to its simple and efficient features. Meanwhile, still some novel composite preparation methods are being studied. For instances, stir casting technology is used to disperse copper particles in aluminum matrix to prepare Al-Cu composites [25,26]. Madhusudan et al. [27] studied the effect of particle composition on composites’ performances by changing copper content in the concentration range of 5 %–15 %. As depicted by results, given the casting and homogenization conditions, the hardness increased with the rise of particle content. Compared with alloys, composite materials were 13 % lower in strength and 15 % lower in ductility. The strength first increased and then decreased with the content rising of the reinforcing agent. Particle concentration and stress concentration serve as main reasons for the decrease of strength. This conclusion has been further verified by the analysis on its microstructure as shown in Fig. 2a–c.

By combining the advantages of aluminum and copper, Al-Cu layered composites exhibit high strength, great corrosion resistance, low density, high conductivity and sound thermal conductivity. Therefore, they have great potential application prospect in auto manufacturing [28]. However, Al-Cu welding is quite challenging due to significant differences in physical and chemical properties between Al and Cu. Gao et al. [29] welded the Al-Cu layered composite plates with vacuum electron beam. In accordance with the research results, the welded beam of aluminum layer was mainly composed of α-Al and Al-Cu eutectic composition; the welded seam center of the copper layer was composed of Al_2_Cu and AlCu intermetallic compounds; the edge was composed of Al_4_Cu_9_, AlCu, and Al_2_Cu intermetallic compounds. The microhardness of parallel aluminum and copper layers did not vary significantly between the substrate and the heat affected zone. The microhardness gradually increased from the substrate to the heat affected zone and then to the Al-Cu interface layer of the welded seam, with the highest hardness reaching 578.1HV. The nanoindentation test showed that the indentation recovery rates were 6.31 %, 10.88 %, and 7.84 % in the upper, interface, and lower parts of the welded seam, respectively. The center of the welded seam showed high elastic modulus and hardness. The maximum tensile strength of the welded joint reaches 64.38 MPa, which is equivalent to 36 % of the substrate strength. The welded seam of aluminum layer showed quasi brittle fracture, while that of the copper layer presented intergranular fracture. These fractures occurred in the area of Al-Cu eutectic structure and Al_2_Cu intermetallic compound. Mao et al. [30] welded the 6061-T6 aluminum with the thickness of 3 mm and T2 copper to study the morphology and corrosion resistance of Al-Cu friction stir welding head. As indicated by the results, Al-Cu joint formed well without cracks or voids. As the corrosion potential of the Al-Cu joint was lower than that of the base material, its corrosion current density was higher than the two base materials. The macroscopic galvanic effect in the joint accelerated its corrosion. The immersion experiment demonstrated that corrosion mainly occurred on the aluminum side. Meanwhile, the most severe corrosion was founded in the heat affected zone, forming relatively large pitting pits. The corrosion product on the aluminum side was Al(OH)_3_/Al_2_O_3_. Al-Cu metal joints with outstanding performance could be developed by optimizing welding process or modifying process parameters. Wang et al. [31] studied the creep behavior and microstructure evolution of thick-plate Al-Cu alloy friction stir welded joints, and optimized the creep age forming process of mechanical components. In accordance with the research, the creep behavior of welds was more sensitive to the increase of external stress in comparison with the substrate. When the stress rose to the average yield strength (140 MPa), the creep deformation and strength of the welded seam improved significantly. In addition, the grain size of the weld nugget zone gradually decreased from top to bottom along the thickness direction, while local strain and dislocation density rose gradually. As a result, the bottom layer of welded seam demonstrated higher creep deformation and steady-state creep rate over creep age forming process. Given the same stress level, the morphology and size distribution of precipitates in the weld nugget area varied from top to bottom after creep age forming process. The upper and middle parts of the welding nugget area contained a large number of fine particles θ″ while larger θ′ usually distributed at the bottom of the weld nugget area. Therefore, exploring appropriate pre-treatment processes to increase the initial dislocation density of the weld can optimize the creep performance of the joint. Ji et al. [32] prepared hypoeutectic Al-30Cu solder with an intermediate frequency induction furnace and analyzed its phase composition, microstructure, melting characteristics, and microhardness properties. Then, the brazing material was used to braze the pure aluminum plates in an argon gas protection furnace. The process parameters were thus adjusted and optimized. The results indicated that the structure of Al-30Cu brazing material was mainly composed of primary α-Al and layered eutectic Al_2_Cu phases. After a 30 min brazing process at 590 °C, pure aluminum brazing can be achieved. The welding output dense structure without defects such as erosion or lack of fusion in the substrate.

The segregation behavior of grain boundaries and the formation of precipitates are key factors in promoting the mechanical properties of Al-Cu alloys. To study the interaction between Cu and Al in Al-Cu alloys, He et al. [33] calculated the segregation energy and charge density distribution of Cu element at the Al∑5(210)[001] grain boundary by making use of density functional theory and first-principles methods. Due to size effects and changes in bonding properties, the formation energy of Cu at grain boundaries is lower and it is prone to segregate towards grain boundary positions with −0.42eV. As verified by high throughput calculations, many metastable phases with negative formation energy, as well as stable ground state structures AlCu_3_, Al_4_Cu_9_, AlCu, and Al_2_Cu were produced in Al-Cu systems at different Cu concentrations. Its elastic modulus and shear modulus were 20 % and 90 % higher than aluminium matrix respectively. In addition, it is an important research method to study Al-Cu composite forming process with finite element models [34]. Ji et al. [35] studied the temperature field evolution of Al-Cu laminated composite materials during casting and rolling process with finite element simulation. It simulated the thermal performance of C18150 copper and 1060 aluminum with JmatPro software, conducted mesh partitioning with Mesh module, and solved the model with Fluent module. Parameters, such as casting speed and pouring temperature, were adjusted and optimized, which was set to check their effects on liquid phase ratio and temperature field. The optimal parameters were thus obtained for the preparation of copper/aluminum/copper laminated composite materials with high strength and sound conductivity. The optimal casting and rolling effect were reached at the optimal casting speed of 1.2 mm/min and pouring temperature of 963 K. Composites with a flat interface and good bonding were produced, including a double interface layer of Al_2_Cu and Al_4_Cu_9_. By optimizing the process parameters, the composite material exhibited excellent tensile strength (201 MPa) and elongation (16 %), and the copper-side conductivity reached 87 % IACS.

With their lightweight, high strength, and excellent heat resistance, Al-Cu reinforcement materials have promising applications [36]. These Al-Cu composites are utilized in various automotive parts, including control components, engine parts, cylinder heads, and wheels. The tests and applications of these AMCs are summarized in Table 1. It is crucial to focus on enhancing efficiency, conducting in-depth research on their working mechanisms, and identifying reliable preparation processes [[37], [38], [39]].

**Table 1:** Mechanical properties of the Al-metal AMCs.

Materials	Hardness (MPa)	Tensile strength (MPa)	UTS (MPa)	Elongation %	Yield strength (MPa)	Application sections	Ref
Al-Cu	578.1	64.38	–	–	–	[57]	[57]
Al-Cu	–	201	–	16	–		[57]
Al-5Cu	510	150	–	–	–		[57]
Al-5Cu	491	130	–	–	–		[57]
Al-10Cu	746	152	–	–	–		[57]
Al-15Cu	844	122	–	–	–		[57]
Al-Cu	71	235	–	9.3	89		[58]
Al-4.4Cu-1.5 Mg	–	–	284/470	6/8.2	177/374		[59]
Al-4.3Cu-1.5 Mg	–	–	293/485	12/9.0	185/399		[60]
Al-4.7Cu-1.5Mg-1.26Ti	–	–	328/428	8/7.4	166/324		[61]
Al-4.57Cu-1.32 Mg	–	–	284/471	6.0/14	177/302		[62]
Al-Mg	–	210.24	–	–	–		[49]
Al-Mg	–	49.5	–	–	–		[51]
Al-Mg	135	–	–	–	–		[52]
Al-5.5Mg-0.2Sc	102 ± 8	–	436 ± 18	26 ± 3	375 ± 15		[53]
Al-Cu-Mg alloy/4wtBPA	66.8	230.5	–	–	–		[63]
Al-Mg-Si+10SiC	77	155	–	23	118		[64]
Al-Mg-Si+2BLA+8SiC	74	146	–	16	110		[64]
Al-Mg-Si+10Al_2_O_3_	75	120	–	12	92		[65]

**Al-Mg Composites:** Common preparation methods include mechanical alloying, vacuum sintering, stir casting and hot-pressing composite [[40], [41], [42], [43]]. It is crucial to study the formation mechanism of Al-Mg alloy in order to further leverage its advantages and improve its thermodynamic and mechanical properties [44,45].

Cai et al. [46] studied the serrated yield phenomenon and deformation distribution of Al-Mg alloy plate specimens with different thicknesses under room temperature loading through three-dimensional digital image correlation technology. Portevin-Le Chatelier (PLC) fringes were observed in the strain field along the stretching direction. The deformation time domain analysis showed that PLC fringes caused a sudden increase in local strain in the stretching direction and corresponding sudden changes in out of plane displacement in the same area. Wang et al. [47] prepared Al-Mg layered composite plates by hot rolling, analyzed the influence of process parameters on the morphology and bonding strength of the composite interface, and studied its bonding mechanism. Results showed that the composite board had a good bonding interface in the temperature range 430 °C–450 °C and a reduction rate of 35 %–50 %. Due to the influence of the interface diffusion layer, the bonding strength reached its peak (∼72.57 N/mm^2^) at a temperature of 450 °C and a 45 % reduction rate. The diffusion dissolution layer at the Al-Mg interface was mainly composed of Mg_2_Al_3_, Mg_3_Al_2_ and MgAl, and its distribution and content were directly affected by the heating temperature. Zuo et al. [48] used Al-Mg alloy sheets as raw materials and prepared Al-Mg micro laminated composite materials with vacuum hot-pressing sintering technology. The impact of the layer thickness ratio on the microstructure (such as structure, element distribution, and crack propagation) and the properties (including compression, tension, and strength ratio) of composite materials was examined. The results showed that composite materials had good bonding performance and the mechanical properties followed the trend of first rising and then falling. Three layers contained in the aluminum magnesium diffusion layer: Mg_17_Al_12_ was on the magnesium rich side, Mg_2_Al_3_ in the middle, and the solid solution layer on the aluminum rich side, with magnesium dissolved in aluminum. Optimal performance of composites was attained when the thickness ratio of the aluminum magnesium layer is 1:1.5, with a flexural strength of 159 MPa, a specific strength of 7.4 × 10^4^ N m/kg, and a compressive strength of 310 MPa. In composites, the accumulation of impurity elements in the Al-Mg diffusion layer is one of the reasons for crack generation and propagation. Therefore, eliminating the influence of harmful elements at the interface is an effective method to enhance the performance of Al-Mg micro laminated composites. Geng et al. [49] used Al/Mg/Cu as raw material and prepared Al-Mg micro laminated composite materials with vacuum hot-pressing sintering technology, so as to study the effect of different layer thickness ratios on the structure and properties of the materials. In the Al-Mg diffusion layer, AlCu_3_ is on the aluminum side, AlCu_4_ in the middle, and a solid solution layer on the magnesium rich side, with copper dissolved in magnesium. With the increase of thickness ratio of aluminum/magnesium layer, the mechanical properties of the material first increase and then decrease. Optimal performance of materials would be attained when the thickness ratio is 1:1.5, with a flexural strength of 365.58 MPa, specific flexual strength of 149.2 × 10^3^ N m/kg, tensile strength of 210.24 MPa and specific tensile strength of 87.4 × 10^3^ N m/kg. The stress distribution and overall material performance were optimized by the alternating toughness and brittle phase structure over material deformation and failure process. Micro laminated composites fully remedy the defects of individual metal elements and integrate their advantages [50].

In response to the current lightweight requirements of the auto industry, the design and manufacturing of Al-Mg composites have become a research focus in the new technology materials and information age. Integrating the corrosion resistance of aluminum with the high specific strength, low density, outstanding damping and vibration reduction performance, and good rigidity of magnesium into the laminated materials not only saves energy and resources, but also reduces product weight and optimizes product performance. Yang et al. [51] prepared Al-Mg bimetallic materials with solid-liquid composite casting method, with aluminum alloy as liquid casting metal and magnesium alloy as solid substrate. The effect of casting temperature of aluminum alloy solution on the interface structure, phase composition, and mechanical properties of composite materials was analyzed. The results indicated that as the temperature of aluminum alloy liquid casting rose up, the width of the transition zone at the interface of the composites increased from 360 μm to 1120 μm and the volume fraction of intermetallic compounds grow accordingly. While in the shear testing process, the interface transition zone was prone to fracture. When the liquid casting temperature of aluminum alloy fell between 660 and 700 °C, a good metallurgical bonding interface of bimetallic materials can be obtained; the interface can be divided into five areas, including the aluminum alloy substrate area, the magnesium alloy substrate area, and the aluminum alloy side mainly composed of α(Al) and Al_3_Mg_2_ phases, magnesium alloy side mainly composed of α-Mg and Mg_17_Al_12_ phases and transition area mainly composed of Mg_17_Al_12_ and Al_3_Mg_2_ phases. The AlMg_4_Zn_11_ phase found in the intermediate transition zone could effectively enhance the bonding performance of composites. When the casting temperature of the aluminum alloy liquid reach 680 °C, the interface strength of composites reached its maximum value, with a shear strength of 39 MPa and a tensile strength of 49.5 MPa. Chen et al. [52] achieved metal bonding between aluminum liquid and cast magnesium alloy through solid-liquid composite method, produced Al-Mg layered composite, and thus solved the problem of poor heat and corrosion resistance of a single magnesium alloy. Diffusion reaction was the main occurred in the intermediate layer of the interface. It was composed of Mg, Al, Mg_2_Al_3_, and a small amount of Mg1_7_Al_12_, with a peak microhardness being up to 135 HV.

**Fig. 3:**
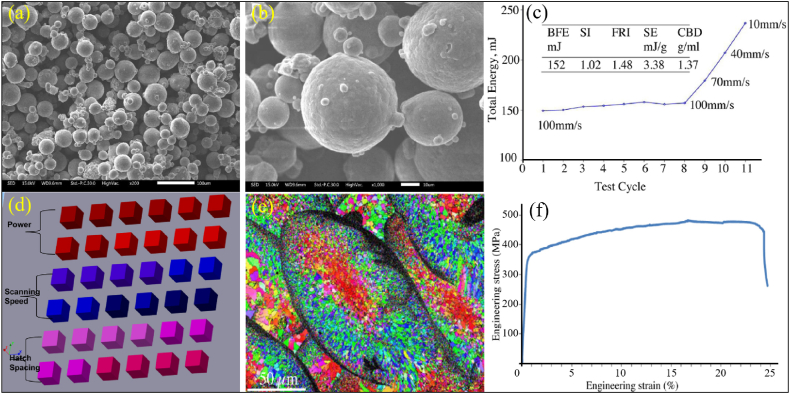
Morphologies (a, b) and rheological behaviors (c) of Al-5.5Mg-0.2Sc powder, (d) Design combination of laser power, scanning speed, and hatch spacing, (e) Electron Back-Scattered Diffraction images of the structure, (f) Engineering tensile stress-strain curve of Al-5.5Mg-0.2Sc sample [53].

The additive manufacturing process could not only construct complex structural components, but also produce parts that are more robust and durable than those manufactured by other existed methods. Qbau et al. [53] prepared and studied a new Al-5.5Mg-0.2Sc material through selective laser melting technology (Fig. 3). Most of raw material powders were spherical in shape and had good flowability (Fig. 3a and b). Each melt pool contained micron-level ultrafine structural bundles in the printed state, exhibiting high mechanical strength (450 MPa) and high ductility (26 %) (Fig. 3c–f).

These Al-Mg composites can be used in automotive components such as transmission cases, connecting rods, rocker arms, bearings, tappets, power transmission cables, driveshafts, automotive bodies, brake discs, and crankshafts. Various tests and applications are summarized in Table 1 [[54], [55], [56]].

### 2.2 Aluminum inorganic composites

**Al-Si alloy** is mainly composed of aluminum and silicon, with a small amount of alloy elements such as copper, magnesium, and titanium added to improve strength. This series of materials exhibit good fluidity, low density, linear shrinkage, high wear resistance, and high corrosion resistance. At present, Al-Si composites are mainly prepared through methods of mechanical alloying, powder metallurgy, high energy ball milling, liquid-phase diffusion, laser melting, and melting reactions [[66], [67], [68], [69], [70]]. Mechanical alloying, known for its high forming efficiency and strong mechanical properties, is suitable for large-scale production; meanwhile, the melting reaction represents an innovative approach to producing Al-Si composites. This method introduces reactants into molten aluminum and silicon to trigger chemical reactions, so as to produce Al-Si composites with strong interfacial bonding and high corrosion resistance. Therefore, it is applicable for the production of composites with high performance. A series of progress have been achieved in the process optimization. Feng et al. [71] solved the problems of long processing time, low thermal energy utilization, and high metal loss in the traditional Al-Si alloy smelting process. A novel energy-saving short process smelting technology was studied, which optimized the smelting furnace and alloy element addition process to secure the practical application of Al-Si composite in production. As the homogenization and refinement of aluminum alloy was realized, this method improved the quality of aluminum alloy, reduced energy consumption by 41 %, saved metal loss by 28.8 %, and improved production efficiency by 17 %. In addition, Tan et al. [72] investigated the effect of different silicon contents on the microstructure and properties of heat-treated Al-Si casting alloys. As shown by results, the precipitates in Al-6%Si alloy were mainly generated along grain boundaries with uneven distribution. A rod-shaped morphology with sharp edges was presented. With the increase of silicon content, the proportion of precipitated phases rose up significantly, leading to larger particle size and the presence of some partially dissolved coarse block like primary silicon. In Al-Si alloys, there are mainly two types of precipitates: one is the long needle shaped AlSiFe phase, and the other is the bright and coarse AlSiMnFe phase. The former has a high surface energy and sufficient vacancy energy to absorb Mn atoms, which spontaneously transforms into a more stable block like AlSiMnFe phase during solidification. This process consumes a large amount of Mn, resulting in a decrease in the Mn/Fe value in the precipitated phase. With the increase of silicon content, the tensile strength of the sample significantly improves, but the ductility and impact resistance do not upgrade correspondingly. In terms of the casting temperature of Al Si alloy, Shi et al. [73] cast Al-Si alloy at 650 °C, 680 °C, and 720 °C, respectively. It was found that with the rise of temperature, cooling speed falls down. As a result, grain size and secondary dendrite spacing rose up and the material performance thus degrades.

**Fig. 4:**
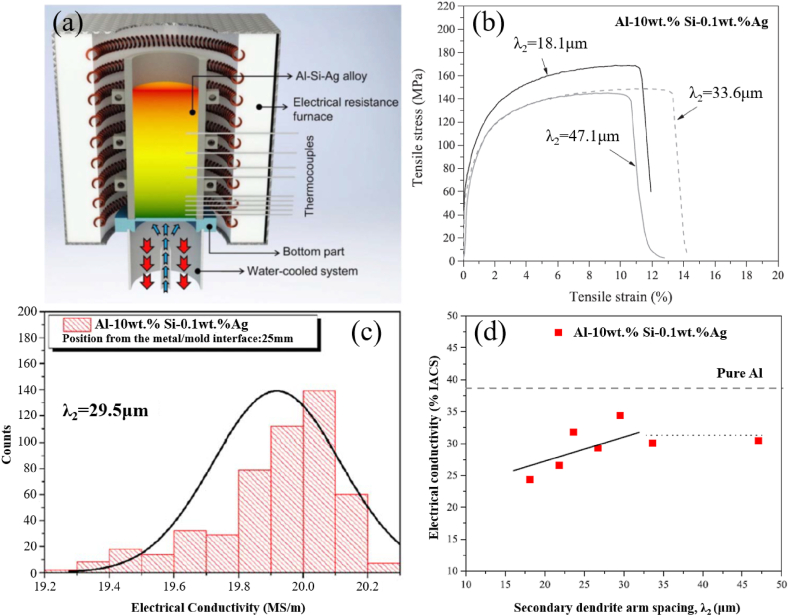
Experimental setup for the study of solidification (a), tensile stress-strain curves of the specimens extracted at different positions from the cooled bottom of the castings Al-10 wt% Si-0.1 wt% Ag (b), Typical histograms showing the electrical conductivity data obtained for the ternary Al-10 wt% Si-0.1 wt% Ag alloy (c). %IACS electrical conductivities of the (a) Al-10 wt% Si-0.1 wt% Ag (d) [75].

**Fig. 5:**
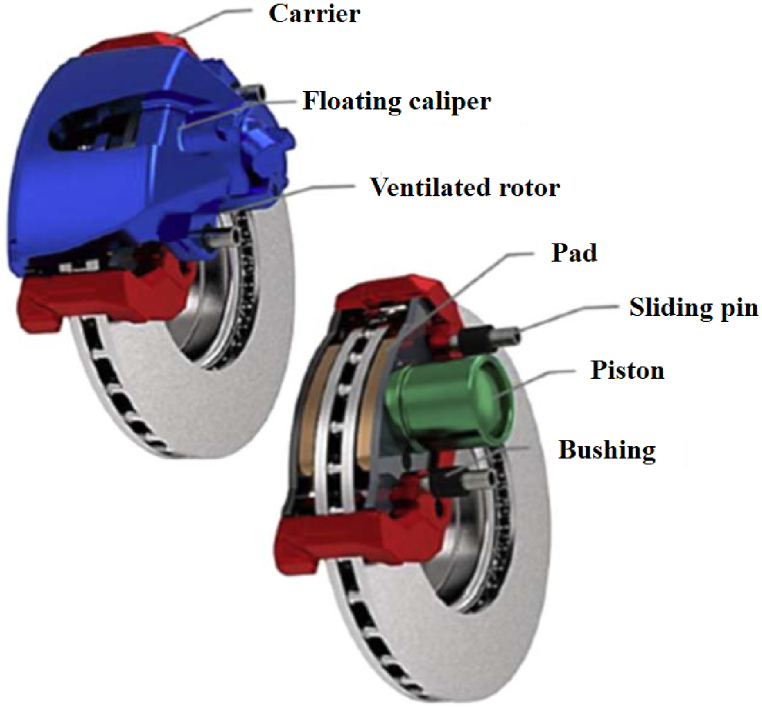
Assembly of a brake disc system [76].

Light weight and system efficiency are hot topics in the auto industry in recent years, and Al- Si alloy material is one of the important research directions [74]. Gomes et al. [75] added Ag of 0.1 and 2.0 wt% to high Al-10 wt%Si alloy to evaluate its microstructure, conductivity, and tensile properties (Fig. 4a–d). In accordance with results, Al-10 wt%Si-0.1 wt%Ag alloy samples featured the strength of 160 MPa, elongation of 12 %, conductivity of 34%IACS. Such indicates that the application prospects of this material in auto industry is promising. In addition, Al-Si composites also have broad application prospects in replacing traditional iron/steel brake discs (Fig. 5). The introduction of Si element further optimizes the thermal performance and wear resistance of materials [[76], [77], [78]]. Some of the tests, including hardness, tensile strength, ultimate tensile strength (UTS), elongation, and applications such as water-cooled cylinder blocks, brake rotors, shafts, catalytic converters, automotive connecting rods, automotive brake discs, and high-speed rotating parts, can be found in Table 2 [[79], [80], [81]].

**Table 2:** Mechanical properties of the Al-nonmetal AMCs.

Materials	Hardness (MPa)	Tensile strength (MPa)	UTS (MPa)	Elongation %	Application sections	Ref
Al-6Si		97.26		5.2	Water-cooled Cylinder blocks, Brake rotors, Cylinder liner, Cylinder heads, Brake drums, Catalytic convertor, Accessories, Intake manifolds, Valve covers, Piston, Pins, Disc brake, Gears, High-speed rotating parts	[72]
Al-12Si		163.23		5.6		[72]
Al-Si				4		[73]
Al-10Si-0.1Ag		160		12		[75]
Al-Si	106.6	213.2		8.65		[84]
Al-Si ZL114A	80	300				[105]
6061Al-2SiC-2B_4_C	45.8	128.24		7.53		[106]
6061A1-40SiC			1492	0.8		[107]
1199A1-25SiC			382			[108]
2009A1-15SiC			548	1.6		[109]
2009A1-SiC			537	9.3		[110]
6092A1-17.5SiC			490	6		[111]
Al 2024-10SiC	87	265		18.2		[112]
Al 2024-10SiC-10FA	95	293		11.9		[112]
Al356-10SiC	100	146				[113]
Al-Al_2_O_3_-Gr	49.97	126		17.11		[114]
2009A1-1CNT			560	10		[115]
Al-0.3 graphene			280	9.5		[115]

The surface quality of mechanical parts directly affects the quality of auto parts. The weariness, fatigue, and corrosion of mechanical components often derive from the surface [82]. Jiao et al. [83] constructed a highly corrosion-resistant superhydrophobic surface structure on AP2 Al-Si alloy by chemical etching, with the surface contact angle reaching 157° and the rolling angle being 5.64°. As this structure enhanced the anti-corrosion performance significantly, it can be used for the structural material of exterior trim.

To further investigate the internal structure and properties of Al-Si composites, Tao et al. [84] used Image Pro Plus to analyze the effect of cryogenic treatment on the microstructure and mechanical properties of die cast Al-Si alloys. Results showed that cryogenic treatment can effectively improve the mechanical properties of die cast Al-Si alloys. As the processing time prolongs, the enhancement effect of cryogenic treatment on the mechanical properties of the alloy first increases and then weakens. In comparison with the original cast alloy, after 12 h of cryogenic treatment, mechanical properties of the material were optimized to the greatest extent, with a tensile strength of 213.2 MPa (an increase of 15.4 %), an elongation of 8.65 % (an increase of 36.2 %), and a hardness of 106.6 HV (an increase of 18.1 %). Further prolonging the cryogenic treatment time will lead to the slow growth of α-Al phase and eutectic Si phases and thus cause the decrease of mechanical performance. Deep cryogenic treatment effectively refined the alloy α-Al phase, improved the size and morphology of eutectic Si and iron containing phases, playing a role in refining the structure and strengthening dislocations.

The application of computational materials and numerical simulation techniques, such as simulation of grain nucleation and growth simulation, pore formation and growth, and simulation of dendritic morphology, has played an important role in the study of microstructure during the solidification process of Al-Si alloys [85]. The simulation at the nanoscale presents a systematic understanding of the evolution of the organization at the atomic level, enabling people to clearly understand the atomic motion trajectory and thus explain the macroscopic phenomena during the solidification process of Al-Si alloys. Structure determines performance, while the migration of atoms or electrons leads to the structural changes in composition. Therefore, nanoscale simulations can not only reveal the patterns of microstructure changes during the solidification process of Al-Si alloys, but also have practical guiding significance for defect repair, product design, and optimization in experimental or engineering manufacturing processes. Nanoscale simulation has become an important direction for the development of numerical simulation technology [86].

**Al-C composites** have great potential in the lightweight, fire resistance, flame retardancy, seismic resistance, and corrosion resistance of new-type automobiles. Common practices include adding granular or fibrous carbon based materials to aluminum alloys to improve the hardness, strength, tensile strength, tensile modulus, heat resistance, wear resistance, and thermal conductivity of composites [87]. At present, domestic and foreign researchers have conducted extensive explorations in the preparation process, performance testing, and application research of carbon reinforced aluminum matrix composites, and significant results have been achieved. The main preparation methods include powder metallurgy, solution impregnation, and mechanical alloying, which involves key steps such as material selection, pretreatment, mixing, forming, and heat treatment.

Currently, carbon has become an ideal choice for high-temperature erosion materials, due to its advantages of low density, strength maintenance with increased temperature, high thermal conductivity, low coefficient of thermal expansion, and good resistance to high-temperature erosion [88,89]. Therefore, Al-C composites are important refractory materials that can withstand harsh environments and directly affect the service life of components. Designing nanocarbons (such as flake, spherical, and tubular nanocarbons) as carbon sources is also crucial [[90], [91], [92]]. Al-C refractory material is a commonly used material for high-temperature functional components in continuous casting. By optimizing the parameters of fabricating process, refractory materials exhibit specific carbon inherent structure, derived carbon structure, and in-situ generated ceramic phase structure at a certain temperature. This microstructure control method enhances the mechanical and thermal shock performance of materials, enabling Al-C functional components to meet the development requirements of continuous casting technology. It, therefore, has important application value in meeting the requirements of automotive appearance and performance of various components.

To improve the thermal strength and stability of Al-C materials, the sintered Al-C skateboards were developed. Thus, the thermal strength and operational safety of the materials were significantly improved through high-temperature sintering under a reducing atmosphere. As the carbon content in Al-C skateboards decreases, the thermal shock resistance of Al-C materials deteriorates accordingly. Tamura et al. [93] proposed the concept of “nanoscale matrix structure” to reduce carbon content while ensuring material strength and thermal shock resistance. The strength and thermal shock stability of the material was comprehensively improved through the adjustment of the nanoscale structural matrix inside the construction material and the utilization of the absorption and buffering of its thermal stress. At present, micro/nano carbon sources are mainly used to replace traditional flake graphite, including nano carbon black (CB), multi walled carbon nanotubes (MWCNTs), graphene nanosheets, and expanded graphite [94]. Nano carbon black is mainly filled in the gaps between Al-C refractory matrix particles, exhibiting its spherical elastic structural characteristics during the thermal shock process of the material. It is used to buffer the thermal stress generated by the expansion of oxide particles and improve the material's thermal shock stability [95]. As nanomaterials with high specific strength, MWCNTs and graphene nanosheets play a role in the fracture process of Al-C refractory materials through mechanisms such as crack bridging, pulling out, and deflection, enhancing the strength and toughness of the materials [96]. In addition, expanded graphite forms a unique wormlike structure through intercalation and the layered structure of expanded flake graphite. When subjected to thermal stress generated by the expansion of the matrix oxide, its structure can effectively absorb and buffer thermal stress, significantly improving the thermal shock stability of the material [97,98]. During this process, carbon containing casting has always been a material receiving great attention due to the poor performance of carbon materials resulted from high-temperature oxidation and unsound wettability. Kuang et al. [99] prepared Al-C casting materials by taking thermal oxidized graphite, ZrC coated modified graphite, and hydrothermal carbonized biomass carbon materials as carbon sources. The effects of different carbon materials on the porosity, physical properties, phase composition, oxidation resistance, and slag corrosion resistance of Al-C casting materials were studied. The results indicated that the casting material containing thermal oxidized graphite had the highest water absorption, and residual pores would damage the internal structure of materials, leading to the deterioration of casting materials' mechanical properties. Adding ZrC surface modified graphite reduced the water absorption of casting materials and exhibit good oxidation resistance, but it hasd little effect on mechanical properties. The physical properties of casting materials could be improved significantly when biomass carbon materials serving as carbon source. Compared with adding graphite, biomass carbon did not significantly improve the slag corrosion resistance of the sample. In addition, the design of catalysts and additives also played an important role in improving the performance of carbon refractory materials [100].

The lightweight design of automobile bumper is crucial for vehicle safety. In order to achieve lightweight design while ensuring collision safety, Kang et al. [101] replaced the conventional steel material of bumper anti-collision beam with Al-C fiber composite material, designed the thickness of different parts in the bumper and the angle of the carbon fiber layer, defined the thickness of aluminum alloy and carbon fiber, and discussed their impact on collision performance. Collision safety was improved through the optimization of the angle of the carbon fiber layer. Experimental samples were established through Latin hypercube sampling. Besides, an approximate model was built through the least squares method and its accuracy was verified. It optimized the approximate model through multi-objective genetic algorithm, determined the optimal lamination angle of carbon fiber and thus obtained optimized Al-C fiber composite bumper beam. Compared with the bumper made of raw steel materials, the optimized new bumper was 36.497 % lighter with the premise of meeting collision safety requirements. In addition, Jin et al. [102] also analyzed the effective connection mechanism between carbon fibers and Al-Mg light alloys. By examining adhesive techniques, mechanical fastening, friction stir welding, and their related process connection methods, the study identified the microstructure and three primary connection mechanisms: macro anchoring, micro mechanical embedding, and chemical bonding. Generally, the key to enhancing the performance of hybrid connections is to increase surface roughness, enlarge the contact area, and implement hybrid connection technology [[103], [104]]. The relevant tests and applications are summarized in Table 2.

### 2.3 Aluminum-plastic composites

With the rise of electric vehicles and intelligent automobiles, aluminum-plastic composites emerged as essential materials in design applications. These composites, composed of aluminum alloys and plastics, exhibit a combination of high strength, low density, corrosion resistance, high wear resistance, lightweight properties, safety, and sealing capabilities [116]. They also demonstrate excellent plasticity and processability, allowing for various shapes and specifications through methods like blow molding, extrusion, and rolling. When used in interior trim, such as aluminum-plastic hybrid dashboard beams, they perform well in surface treatment and decoration. As external decorative materials, they exhibit good fire resistance, waterproofing, and thermal insulation. Their impressive fatigue resistance and anti-aging properties make them suitable for harsh environments, prolonging the lifespan of auto parts. Moreover, aluminum-plastic materials are highly recyclable, which helps to reduce raw material and energy consumption in automotive manufacturing, lower production costs, decrease environmental pollution, and enhance the efficiency and stability of power systems. This contributes to improved market competitiveness for automobiles.

**Fig. 6:**
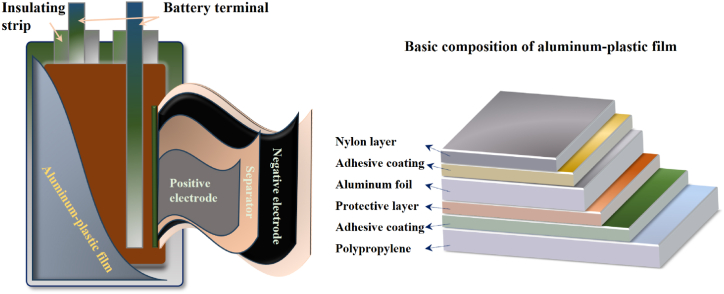
Composition of pouch battery and basic composition of aluminum-plastic film.

In electric vehicles, aluminum-plastic composite film is an important component of the power battery structure (Fig. 6). When aluminum-plastic film serves as the outer packaging material in pouch cells, the energy density can be 5–15 % higher than that of cylindrical and square batteries with steel or aluminum shells. This provides a solution for the high energy density of lithium-ion batteries. Aluminum plastic film composed of nylon, aluminum foil, and polypropylene three-layer films, is the core material of pouch cells. Aluminum foil features excellent corrosion resistance. Plastic demonstrates good sealing performance, and are also characterized by lightweight, insulation, heat resistance, impact resistance, moisture resistance, and pollution prevention. Technical requirements for aluminum-plastic film in lithium-ion batteries are as below: good puncture resistance and pollution resistance, heat sealing layer and internal film with high temperature resistance to avoid short circuit and high flexibility, as well as good cold stamping formability. Therefore, aluminum-plastic composite materials can effectively protect the internal materials of batteries and extend their service life. Although the structure of aluminum-plastic film is simple, its production technology is much more complex than other battery materials. At present, the research on aluminum foil treatment process and composite adhesive formula is still insufficient in our country. Therefore, in-depth research is great significance in ensuring stable long-term operation of batteries and meeting the high energy density requirements of new energy automobiles.

**Fig. 7:**
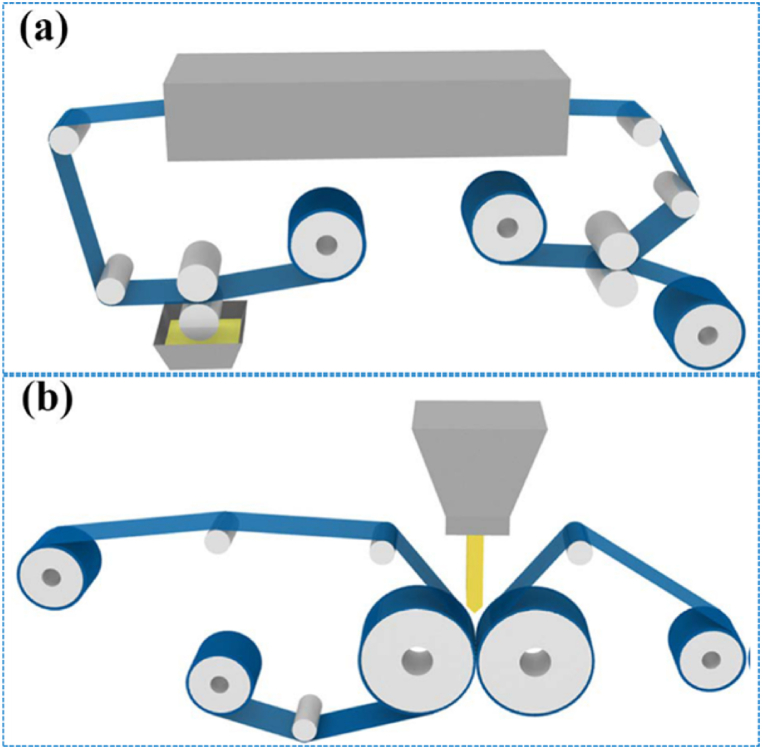
The preparation process of aluminum plastic film: (a) Dry lamination; (b) Hot-press lamination [116].

Currently, the main production processes for aluminum-plastic films are dry and hot methods (Fig. 7a and b). In the dry process, aluminum and polypropylene are bonded by adhesive and synthesized under dry pressure. The thermal method involves using porous foaming materials to bond aluminum and polypropylene, followed by slow heating and pressing to synthesize. Dry process aluminum-plastic film has the advantages of good forming performance, excellent short circuit resistance, few surface defects, fast production speed, simple process, and low cost. However, its peel strength is low and special adhesives are required to ensure performance such as electrolyte resistance and water resistance. Meanwhile, the aluminum-plastic film produced by thermal method has a strong combination of aluminum and polypropylene layers, enhancing its resistance to electrolyte expansion and internal surface layer peeling. However, differences in material shrinkage coefficients can lead to inward curling during the cooling process in thermal methods, resulting in a poor appearance and cutting performance. Heating would make the material brittle, resulting in poor tensile performance, short circuit resistance, and relatively complex production processes. In addition to these methods, post production processes such as cutting, printing, and forming are equally important to ensure that the final aluminum-plastic film meets the requirements. Throughout the entire process, it is necessary to strictly control the process parameters at each stage to ensure the stability and consistency of the quality of the aluminum-plastic film.

**Fig. 8:**
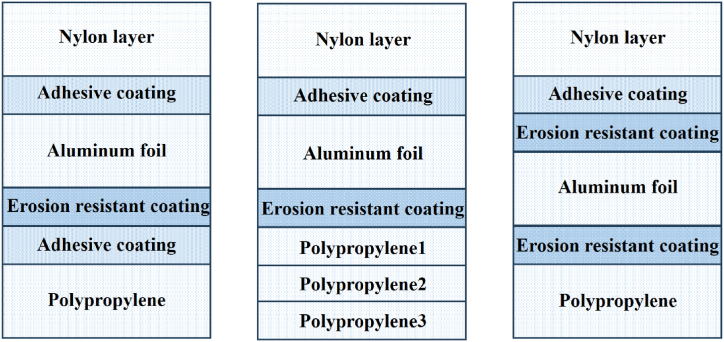
Diagrams of the structure for different aluminum-plastic films.

The manufacturing process of aluminum-plastic film raises requirements on the compatibility between the binder and the substrate. Improper coordination will lead to interface failure and the inability to exert the material's function (Fig. 8). Common failure modes include interface damage, substrate damage, and cohesive failure. Interface damage is mainly presented as interlayer delamination, leading to inconsistent deformation and premature failure of the substrate. The deteriorated interlayer delamination strength is usually caused by improper surface treatment of aluminum foil, nylon or polypropylene layers [117]. Substrate damage refers to the phenomenon that mechanical properties of the substrate being insufficient to withstand deformation loads and strains, resulting in fracture. Compared with nylon and polypropylene layers, aluminum foil has lower ductility and is most prone to failure. Cohesive failure refers to the damage to the adhesive layer that occurs between substrates. Poor adhesion and corrosion resistance between the aluminum foil and polypropylene interface is the major challenge in preparing aluminum-plastic film. In this case, polypropylene tends to peel off the aluminum foil and affect the heat-sealing performance. Therefore, it is significant for the interface structure and safety design of batteries by conducting research on the application performance of aluminum foil, such as the influence of surface morphology and surface treatment on the aluminum-plastic composite performance, to understand the peeling mechanism of aluminum-plastic film under extreme conditions [118]. In order to enhance the heat-sealing performance and corrosion resistance of aluminum-plastic films, Shi et al. [119] proposed a new method for preparing hot aluminum-plastic films based on polypropylene. In comparison with the conventional wet and dry methods, this method solved the problems in direct polypropylene coating and hot-pressed aluminum foil surface by depositing a nano metal anti-corrosion coating on the surface of aluminum foil to bond polypropylene and aluminum foil. The research results indicated that the nano coating process could increase the surface roughness of aluminum foil and expand the contact area between aluminum foil and polypropylene. In accordance with the heat-sealing experiment, the primary sealing strength of the polypropylene thermal method aluminum-plastic film exceeded 140 N/15 mm, and the secondary sealing strength was still well maintained after soaking in the electrolyte. This aluminum-plastic film has good heat-sealing performance and corrosion resistance, demonstrating potential application value in the field of power batteries. In terms of process, Yuan et al. [120] produced aluminum-plastic film products with composite dry process, which does not need production conditions required by of aluminum-plastic film thermal methods, such as high temperature and high pressure. As indicated results, the preparation of composite aluminum-plastic film effectively maintained the deep drawing performance and product appearance of the aluminum foil, with a normal peel strength of 17.6 N/15 mm, an electrolyte peel strength of 14.2 N/15 mm, and a maximum deep drawing of 7.3 mm. As it solves the problem of electrolyte corrosion resistance of aluminum-plastic films, this method has potential application.

### 2.4 Mechanism

**Fig. 9:**
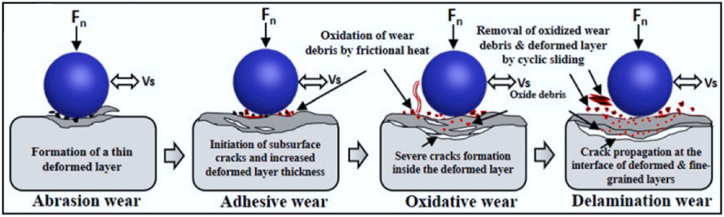
Schematic of the wear mechanism as the sliding time increases [123,124].

During automobile operation, materials constantly face failure. Generally, in the automotive sector, the failure process of a material can be divided into three stages in the model, which include elastic deformation stage, body damage stage and localization damage stage [121]. These stages correspond to several fundamental wear mechanisms, including adhesive wear, abrasive wear, corrosive wear/oxidative wear, and surface fatigue wear/delamination wear (Fig. 9) [122]. Adhesive wear occurs due to interactions between rubbing surfaces, while abrasive wear happens when hard surfaces or particles create grooves in softer materials. Corrosive wear involves chemical reactions between contact surfaces and the environment, leading to reaction layers that are eventually worn away by mechanical interactions. Surface fatigue wear, common in rolling contacts, manifests as pits or flakes on surfaces, resulting from repeated high contact stresses [123]. Critical factors such as load, abrasive element size, number of abrasive particles, and material toughness are key adjustable variables.

Therefore, to address the low strength of pure aluminum, various approaches have been implemented, including the development of AMCs by combining aluminum with copper, magnesium, silicon, and carbides. As discussed in this review, several AMCs are detailed in Table 1, Table 2. Incorporating these elements enhances aluminum's properties, such as hardness, tensile strength, ultimate tensile strength, and elongation. The findings indicate that the mechanical properties of AMCs surpass those of unreinforced materials. Therefore, understanding the reinforcement process and strengthening mechanisms is crucial for further analysis of AMCs material and their applications in the automotive sectors.

**Fig. 10:**
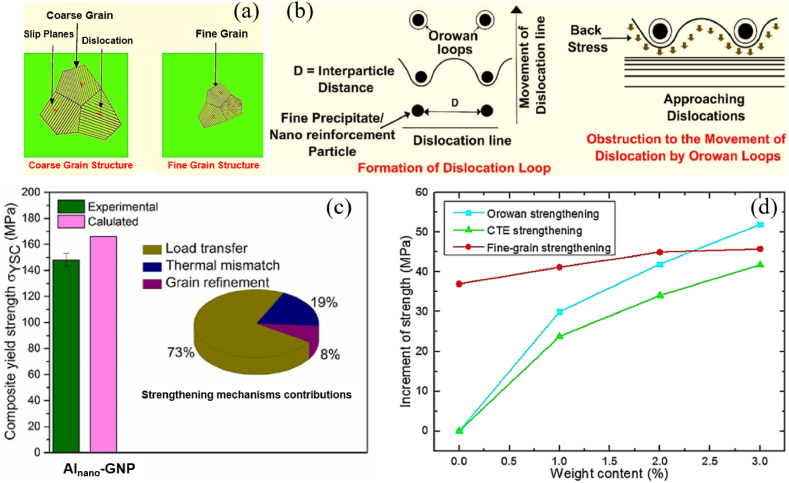
Schematic representation of strengthening *via* grain refinement (a). Orowanian strengthening mechanism (b). (c, d) Contribution of different strengthening mechanism [[126], [127], [128]].

Currently, several mechanisms contribute to the strength of AMCs, including grain refinement strengthening (Hall-Petch relationship, σ_н-p_), dislocation strengthening (Taylor equation, σ_Taylor_), thermal expansion strength (coefficient of thermal expansion, σ_*CTE*_), dispersion strengthening (Orowan mechanism, σ_or_), and load transfer (σ_L-T_) (Fig. 10a and b) [124,125]. Grain refinement is the most universal and effective method for strengthening AMCs, leveraging the “smaller is stronger” effect, which is typically achieved through the Hall-Petch effect related to grain size. While grain refinement enhances the strength of the matrix, it can simultaneously weaken the thermal expansion mismatch strengthening. Often, the increase in strength of AMCs comes at the expense of ductility and toughness. Furthermore, high reinforcement content, especially with nanopowders, can lead to agglomeration and defects like micro-cracks. Therefore, homogeneous nanoparticles within AMCs are crucial for effective reinforcement. Additionally, an integrated microstructure with a 3D network can be formed, resulting in a continuous AMCs net configuration that exhibits excellent stiffness, hardness, and thermal properties. Moreover, coupling two or more types of elements with aluminum in AMCs shows superior performance compared to single-reinforced composites. These elements have a significant synergistic reinforcement effect on the composite's properties, enhancing wear and chemical resistance. During the reinforcement process, one or more mechanisms will contribute to the strength of the properties. The main contributing mechanism varies across different composites [[126], [127], [128]]. Fig. 10 c, d illustrates the influence of different strengthening mechanisms within various AMCs.

Additionally, the joining of dissimilar metals offers numerous advantages, with intermetallic phases forming due to dynamic recrystallization. In some cases, intermetallic phases like AlCu, Al_2_Cu, Al_3_Cu_4_, and Al_4_Cu_9_ in Al-Cu alloys can enhance the strength and ductility of welded joints [129]. In aluminum carbide composites (such as CNT, graphene, or graphite), the intermetallic compound Al_4_C_3_ forms through interfacial reactions, facilitating effective load transfer from the matrix to Al_4_C_3_ and increasing material hardness [130]. It has also been observed that friction and wear decrease in the presence of Al_4_C_3_. However, intermetallic compounds sometimes have detrimental effects on strength. The formation of these phases on surfaces may lead to poor weld surface quality and aesthetics [131]. In addition, variations in the amount of intermetallic phases can cause uneven surfaces and introduce vacancies and interstitial defects during welding [132]. This can result in defective joints and the emergence of cracks on the weld surfaces, negatively impacting the strength and corrosion resistance of the weld. To overcome these challenges, several strategies should be developed, such as applying anti-corrosion coatings and utilizing surface treatments to mitigate defects in intermetallic phases [133].

## 3 Summary and prospect

In summary, AMCs have demonstrated significant potential for use in the automotive sectors. Their advantages are particularly evident in automobile decoration, power systems, and main components. Above all, AMCs offer an opportunity to reduce weight by replacing heavier materials such as steel. This review summarizes and analyzes the preparation methods, wear mechanisms, performance enhancement strategies, strengthening mechanisms and intermetallic compounds impacts of AMCs, discussing key factors that influence the development of new AMCs. However, there are still critical limitations regarding their application, including: (1) The manufacturing of AMCs requires high-precision equipment and technology. (2) New recycling methods need to be explored to minimize irreversible environmental impacts. For example, the production of aluminum-plastic films generates a large amount of solid waste and wastewater, causing serious environmental issues. Specifically, aluminum foil production produces significant amounts of chlorofluorocarbons, which are harmful to the atmosphere. Additionally, the composite nature of aluminum foil and plastic complicates recycling and increases costs, making it difficult to repurpose aluminum-plastic films and resulting in resource waste. Therefore, sustainable recycling of aluminum-plastic films requires our attention. (3) There is still need for improvements in strength, elastic modulus, and abrasion resistance of AMCs through the development of new fillers and surface treatment technologies. (4) Welding technology for AMCs is relatively complex and challenging to operate. (5) The cost of AMCs is a critical factor driven by consumer demand, influencing their use in automotive components. Therefore, producing AMCs at a low cost is essential. Typically, AMCs made through powder metallurgy, stir casting, squeeze casting, and spray casting are moderately priced, making them suitable for applications like fasteners, cylinders, valves, high-hardness materials, high-temperature materials, and small circular items. In-situ processing and ultrasonic-assisted casting are more expensive methods used for producing sheets, blades, vane shafts, and structural components. (6) Additionally, the relationship and influence patterns between material microstructure and properties require further exploration.

At last, the application of AMCs in automobiles presents significant research potential. Studying the new structures of AMCs and the underlying mechanisms that enhance their mechanical, thermal, and other properties is highly important. Conducting in-depth research on AMCs to optimize their performance would drive technological innovation and consequently accelerate growth in the automotive industry and the new energy sectors.

## Data and code availability statement

Data included in article is referenced in the article.

## CRediT authorship contribution statement

**Xiaodong Wu:** Writing – review & editing, Writing – original draft, Funding acquisition. **Wenkang Zhang:** Investigation.

## Declaration of competing interest

The authors declare the following financial interests/personal relationships which may be considered as potential competing interests:Wu Xiaodong reports financial support was provided by 10.13039/100014472Scientific Research Foundation of Hunan Provincial Education Department. If there are other authors, they declare that they have no known competing financial interests or personal relationships that could have appeared to influence the work reported in this paper.
